# Clinical features and laboratory indicators of dengue infection in China: a retrospective study of adult patients in a hospital of traditional Chinese medicine

**DOI:** 10.3389/fmed.2025.1624554

**Published:** 2025-07-07

**Authors:** Qilong Nie, Mingyang Li, Qiuyan Liang, Jian Ren, Tong Li, Wenya Peng, Cuifen Luo, Xiaoai Mo, Xiaojun Ma, Jianhong Li, Kaiping Jiang

**Affiliations:** ^1^The Eighth Clinical Medical College, Guangzhou University of Chinese Medicine, Foshan, Guangdong, China; ^2^Foshan Hospital of Traditional Chinese Medicine, Foshan, Guangdong, China

**Keywords:** dengue infection, liver enzymes, inflammatory markers, tissue damage, retrospective observational study

## Abstract

**Background:**

Dengue is an arboviral disease caused by the dengue virus, primarily transmitted by mosquitoes in tropical and subtropical regions. Despite preventive measures, the incidence and mortality of dengue remain significant. While the acute phase of infection often presents with mild, self-limiting symptoms and may mimic other undifferentiated febrile illnesses, the risk of mortality is particularly high during the acute phase of secondary infections, which are associated with more severe clinical manifestations. Liver dysfunction has been strongly linked to the severity of the disease, and it plays a critical role in determining patient outcomes.

**Methods:**

This retrospective observational study was conducted at Foshan Hospital of Traditional Chinese Medicine, including 533 hospitalized dengue patients diagnosed between June and December 2024. Clinical symptoms (e.g., fatigue, headache, muscle pain, dry mouth, rash, nausea) and laboratory parameters (e.g., complete blood count, liver function tests, lactate dehydrogenase, C-reactive protein, procalcitonin) were collected. Patients were categorized into three groups based on liver function test results: non-liver injury (ALT ≤ 40 U/L, AST ≤ 40 U/L), mild liver injury (ALT or AST levels between 40 U/L and 80 U/L), and severe liver injury (ALT > 80 U/L or AST > 80 U/L).

**Results:**

Among the 533 patients, 48.03% were male and 51.97% were female, with the majority (61.35%) in the 51–80 years age range. Common clinical symptoms included fatigue (92.50%), poor appetite (90.99%), dry mouth (62.66%), and headache (52.53%). Significant laboratory abnormalities included leukopenia (63.41%), thrombocytopenia (80.11%), and elevated liver enzymes (AST 84.99%, ALT 52.53%). Stratification based on liver injury severity showed that the severe liver injury group had significantly higher levels of inflammatory markers (CRP, PCT), and tissue damage markers (LDH, CK) compared to the non-liver injury and mild liver injury groups. The severe liver injury group also had a younger median age compared to the other two groups (*p* < 0.05). Additionally, bone joint pain and melena were more frequently observed in the severe liver injury group, highlighting their association with liver injury severity.

**Conclusion:**

Dengue patients commonly present with symptoms such as fatigue, poor appetite, and dry mouth, with laboratory abnormalities including leukopenia, thrombocytopenia, and elevated liver enzymes.

## Introduction

Dengue is an arboviral disease caused by the dengue virus (DENV), one of the most dangerous and common mosquito-borne viral diseases. It is predominantly found in tropical and subtropical regions and affects more than 100 countries worldwide (1). Dengue continues to increase at a faster rate than any other communicable disease, with a significant rise over the past 24 years (2000–2024). The annual incidence is estimated at approximately 100 million symptomatic cases, with an additional 300 million asymptomatic infections. The greatest burden is seen in Asia (75%), followed by Latin America and Africa. In 2024, a record high of 14,361,142 cases and 10,839 deaths were reported from 112 countries to the WHO.^1^

Dengue infection can present with a range of clinical manifestations, from mild fever to severe forms such as dengue shock syndrome (DSS) and dengue hemorrhagic fever (DHF), both of which can be fatal. While these severe forms are more commonly associated with secondary dengue infections (2, 3), they can also occur in primary infections, particularly in certain high-risk groups (4–6). The clinical spectrum of dengue is influenced by various factors, including the patient’s immune response, the serotype of the virus, and the presence of underlying comorbidities (7). Accurate and early diagnosis is essential for effective management, as severe forms of the disease, such as DSS and DHF, can rapidly progress to life-threatening conditions without prompt medical intervention (8, 9).

The pathogenesis of dengue is complex, involving interactions between the virus and the host’s immune system (10). Secondary infections with different serotypes of the virus increase the risk of severe manifestations due to immune enhancement (11). Diagnosis is based on clinical presentation and laboratory tests, such as serological assays, nucleic acid amplification tests, and ELISA assays for detecting the NS1 antigen (12, 13). Despite advances in prevention, including vector control and insect repellents, there is no specific antiviral treatment for dengue. Management remains supportive, with an emphasis on fluid therapy and careful monitoring in severe cases (14, 15).

This study aims to retrospectively analyze and perform descriptive statistical analysis on the clinical features and laboratory indicators of hospitalized dengue patients. The focus is particularly on laboratory results such as complete blood counts (CBC) and liver function tests, with a specific emphasis on liver injury. By reviewing historical patient records, this study seeks to provide a comprehensive description of the clinical characteristics and laboratory findings of dengue patients admitted to the hospital.

## Methods

### Study design and participants

This retrospective study was conducted at Foshan Hospital of Traditional Chinese Medicine from June to December 2024. A total of 533 hospitalized patients diagnosed with dengue fever were included in this study. The inclusion criteria for confirmed cases were in accordance with the Dengue Diagnosis and Treatment Protocol (2024 Edition), developed by the National Health Commission of China and the National Administration of Traditional Chinese Medicine, which include the following (16), a national guideline formulated by the National Health Commission of China and the National Administration of Traditional Chinese Medicine. These guidelines were specifically developed to address the epidemiological and clinical characteristics of dengue within China. The inclusion criteria for confirmed dengue cases were established in line with these guidelines, which take into account both clinical manifestations and laboratory test results specific to the Chinese context, which include the following:

Epidemiological history: The patient has a history of traveling to a dengue-endemic area within the 14 days before the onset of symptoms or has been exposed to dengue cases within 1 month around their residence or workplace.

Clinical manifestations: (1) acute onset: Sudden high fever, fatigue, loss of appetite, nausea, and often accompanied by severe headache, retro-orbital pain, myalgia, and arthralgia. It may also include facial, neck, and chest flushing, and conjunctival congestion. (2) Rash: Between days 3 and 6, patients may develop a congested rash or petechial rash, typically appearing on the limbs, face, and trunk, with itching and no desquamation, lasting 3–5 days. (3) Bleeding tendency: Symptoms include petechiae, gum bleeding, epistaxis, and positive tourniquet test. (4) Severe bleeding: Subcutaneous hematomas, gross hematuria, gastrointestinal, chest, abdominal, vaginal, and intracranial bleeding. (5) Severe organ damage: Symptoms like acute myocarditis, acute respiratory distress syndrome, acute liver damage, acute renal failure, and central nervous system injury. (6) Shock: Features include tachycardia, cold extremities, prolonged capillary refill (>3 s), weak or undetectable pulse, small pulse pressure, or undetectable blood pressure.

Laboratory tests: (1) Leukopenia and/or thrombocytopenia. (2) Dengue IgM antibody positive. (3) Dengue NS1 antigen positive within 5 days of illness onset. (4) Dengue-specific IgG antibody titer increasing fourfold or seroconversion from acute to recovery phase. (5) Isolation of the dengue virus from acute phase samples (blood, cerebrospinal fluid, or tissues). (6) Isolation of the dengue virus from acute phase samples (blood, cerebrospinal fluid, or tissues).

Diagnosis classification: (1) suspected case: Meets one of the following criteria: (a) the first item has an epidemiological history and meets the clinical manifestations. (b) It simultaneously meets the first item of clinical manifestations and the first item of laboratory tests. (2) Clinical diagnosis: Meets one of the following criteria: (a) Meet the first item of suspected cases and simultaneously meet one of the second and third items of clinical manifestations, and also meet the first item of laboratory tests. (b) Meets the criteria of a suspected case and simultaneously meets one of the second and third items of the laboratory test. (3) Confirmed case: It meets the criteria for suspected cases or clinically diagnosed cases, and simultaneously meets one of the fourth, fifth, and sixth items of laboratory tests. (4) Severe dengue: It is a clinically diagnosed case or a confirmed case, and simultaneously meets one of the fourth, fifth or sixth clinical manifestations.

Inclusion Criteria: (1) Confirmed cases of dengue fever infection of all ages diagnosed according to the above guidelines. Exclusion Criteria: (1) Incomplete clinical or laboratory data. (2) Patients with co-infections [e.g., malaria, typhoid fever, or other viral infections including SARS-CoV-2 infection (COVID-19)] or diseases that might interfere with the assessment of dengue severity. (3) Patients with pre-existing chronic liver diseases or other major comorbidities, such as renal failure or cardiovascular diseases, which might confound the interpretation of clinical data.

According to the Dengue Diagnosis and Treatment Protocol (2024 Edition) (16), a high level of IgG antibodies detected within the first week of illness is suggestive of a secondary dengue infection. However, upon reviewing the medical records of all patients included in our study, we found that IgG antibody testing was not performed for any of the cases. As a result, we were unable to reliably differentiate between primary and secondary dengue virus infections.

### Data collection

Data were retrospectively collected from the hospital’s electronic medical records. The following variables were gathered for analysis: demographic data including age and gender; laboratory parameters such as white blood cell count (WBC), hemoglobin (HGB), platelet count (PLT), absolute neutrophil count (ANC), liver function tests (Alanine aminotransferase, ALT, Aspartate aminotransferase, AST, Gamma-glutamyl transferase, GGT, Total bilirubin, TBIL), lactate dehydrogenase (LDH), creatine kinase (CK), C-reactive protein (CRP), and procalcitonin (PCT); clinical symptoms including dry mouth, bitter taste, fatigue, poor appetite, nausea, diarrhea, retro-orbital pain, headache, muscle pain, joint pain, rash, epistaxis, and melena; and clinical outcomes including the time from the onset of symptoms to the detection of liver damage.

However, during the data collection process, information regarding patients’COVID-19 infection history and vaccination status was not recorded in the medical records. Therefore, these potential confounding factors were not included in the analysis. Previous studies have suggested that COVID-19 infection or vaccination could potentially increase the risk of symptomatic dengue infection (17, 18). It is recommended that future studies incorporate these factors to explore their potential impact on the clinical outcomes of dengue.

### Group classification

Patients were categorized into three groups based on their liver function test results. Non-liver injury group: This group included patients with normal ALT and AST levels, defined as ALT ≤ 40 U/L and AST ≤ 40 U/L. A total of 333 patients were included in this group. Mild liver injury group: This group comprised patients with either ALT or AST levels elevated to no more than two times the upper normal limit. Specifically, ALT or AST levels between 40 U/L and 80 U/L were considered as mild liver injury. A total of 113 patients fell into this category. Severe liver injury group: The severe liver injury group consisted of patients with either ALT or AST levels exceeding two times the upper normal limit, which was defined as ALT > 80 U/L or AST > 80 U/L. This group included 87 patients. These groupings were used to assess differences in clinical characteristics, laboratory parameters, and clinical outcomes based on the severity of liver injury in hospitalized dengue patients.

### Correlation analysis

To assess the relationships between WBC, PLT, ALT, and AST, both Spearman’s rank correlation and Pearson’s correlation were calculated within the following three groups: non-liver injury group, mild liver injury group, and severe liver injury group.

Spearman’s rank correlation is a non-parametric method used to assess the strength and direction of association between two variables. It is suitable for data that may not follow a normal distribution and is used to evaluate monotonic relationships, where variables tend to change in the same or opposite direction, but not necessarily in a linear fashion. Pearson’s correlation, on the other hand, measures the strength of a linear relationship between two continuous variables, assuming that the data are normally distributed. The correlation coefficient ranges from −1 to +1, where +1 indicates a perfect positive correlation, −1 indicates a perfect negative correlation, and 0 indicates no correlation. Additionally, to visually represent the relationships between these variables, a correlation matrix heatmap was generated. The heatmap displays the correlation coefficients between the variables, with color intensity and circle size reflecting the strength of the correlation.

### Statistical analysis

Descriptive statistics were used to summarize the demographic, clinical, and laboratory characteristics of the patients. Continuous variables were tested for normality using the Shapiro–Wilk test. For normally distributed data, means and standard deviations (SD) were calculated. Non-normally distributed data were presented as medians and interquartile ranges (IQR). For categorical variables, frequencies and percentages were reported.

To compare the clinical and laboratory characteristics across the three groups (non-liver injury, mild liver injury, and severe liver injury), one-way analysis of variance (ANOVA) was used for continuous variables with normal distribution, and the Kruskal-Wallis test was applied for non-normally distributed continuous variables. For categorical variables, the chi-square test or Fisher’s exact test (when appropriate) was used to evaluate the differences between the groups.

Additionally, pairwise comparisons were conducted for continuous variables using Tukey’s post-hoc test (for normally distributed data) or Dunn’s test (for non-normally distributed data), and for categorical variables, pairwise comparisons were performed using adjusted *p*-values from chi-square tests. Spearman’s rank correlation coefficient was used to assess the relationships between continuous variables where appropriate. All statistical analyses were conducted using SPSS version 26.0 (IBM Corp., Armonk, NY) and R software (version 4.2.1, R Foundation for Statistical Computing). A *p*-value < 0.05 was considered statistically significant.

## Results

### Patient demographics

A total of 533 hospitalized patients diagnosed with dengue fever were included in this study, comprising 256 males (48.03%) and 277 females (51.97%). The age distribution of the patients is presented in Table 1. The majority of patients were aged 51–80 years, accounting for 61.35% (*n* = 327) of the total cases. The largest proportion of patients was found in the 71–80 age group (25.70%, *n* = 137), followed by the 51–60 age group (20.45%, *n* = 109) and the 61–0 age group (15.20%, *n* = 81). Patients younger than 30 years made up only 7.68% (*n* = 41) of the total cases, while those older than 90 years represented just 0.94% (*n* = 5). Regarding gender distribution across age groups, the highest proportion of males was observed in the 71–80 age group (27.34%), while females were most predominant in the same age group, accounting for 24.19%. In the older age groups (≥81 years), the distribution between males and females remained relatively balanced.

**Table 1:** Age distribution of 533 cases of dengue fever [n(%)].

Age	Cases	Males	Females
18–20	14 (2.62)	9 (3.52)	5 (1.81)
21–30	27 (5.06)	14 (5.47)	13 (4.69)
31–40	39 (7.31)	24 (9.38)	15 (5.42)
41–50	49 (9.19)	15 (5.86)	34 (12.27)
51–60	109 (20.45)	43 (16.80)	66 (23.83)
61–70	81 (15.20)	40 (15.63)	41 (14.80)
71–80	137 (25.70)	70 (27.34)	67 (24.19)
81–90	72 (13.51)	39 (15.23)	33 (11.91)
>90	5 (0.94)	2 (0.78)	3 (1.08)
Total	533 (100.00)	256 (48.03)	277 (51.97)

### Clinical features of dengue patients

The laboratory findings revealed significant abnormalities, as summarized in Table 2. Leukopenia was observed in 63.41% of the patients, and thrombocytopenia was present in 80.11%, leukopenia and thrombocytopenia are commonly observed during the later stages of acute dengue infection and may indicate disease severity or progression toward dengue hemorrhagic fever or dengue shock syndrome. Elevated levels of AST and ALT were found in 84.99 and 52.53% of patients, respectively, suggesting liver involvement. Additionally, elevated ANC, GGT, and TBIL were seen in 26.45, 41.46, and 11.63% of patients, respectively.

**Table 2:** Clinical presentation and laboratory abnormalities in 533 cases of dengue fever.

Variable	Total	N (%)
Leukopenia	533	338 (63.41)
Hypoglobinemia	533	82 (15.38)
Thrombocytopenia	533	427 (80.11)
AST increase	533	453 (84.99)
ALT increase	533	280 (52.53)
ANC increase	533	141 (26.45)
GGT increase	533	221 (41.46)
TBIL increase	533	62 (11.63)
LDH increase	533	324 (60.79)
CK increase	345	215 (62.32)
CRP increase	372	206 (55.38)
PCT increase	371	354 (95.41)
Fatigue	533	493 (92.5)
Headache	533	280 (52.53)
Muscle pain	533	226 (42.4)
Bone joint pain	533	80 (15.01)
Poor appetite	533	485 (90.99)
Nausea	533	253 (47.47)
Dry mouth	533	334 (62.66)
Bitter taste in the mouth	533	247 (46.34)
Rash	533	195 (36.59)
Diarrhea	533	210 (39.4)
Hemorrhage	533	12 (2.25)
Melena	533	27 (5.07)

AST, Aspartate Aminotransferase; ALT, Alanine Aminotransferase; ANC, Absolute Neutrophil Count; GGT, Gamma-Glutamyl Transferase; TBIL, Total Bilirubin; LDH, Lactate Dehydrogenase; CK, Creatine Kinase; CRP, C-Reactive Protein; PCT, Procalcitonin.

In terms of clinical symptoms, fatigue was the most common symptom, present in 92.50% of patients. Headache was reported in 52.53% of cases, while muscle pain and joint pain occurred in 42.40 and 15.01% of patients, respectively. Gastrointestinal symptoms such as poor appetite (90.99%), nausea (47.47%). Other notable symptoms included bitter taste in the mouth (46.34%) and rash (36.59%).

### Physiological and biochemical characteristics of dengue patients

**Figure 1:**
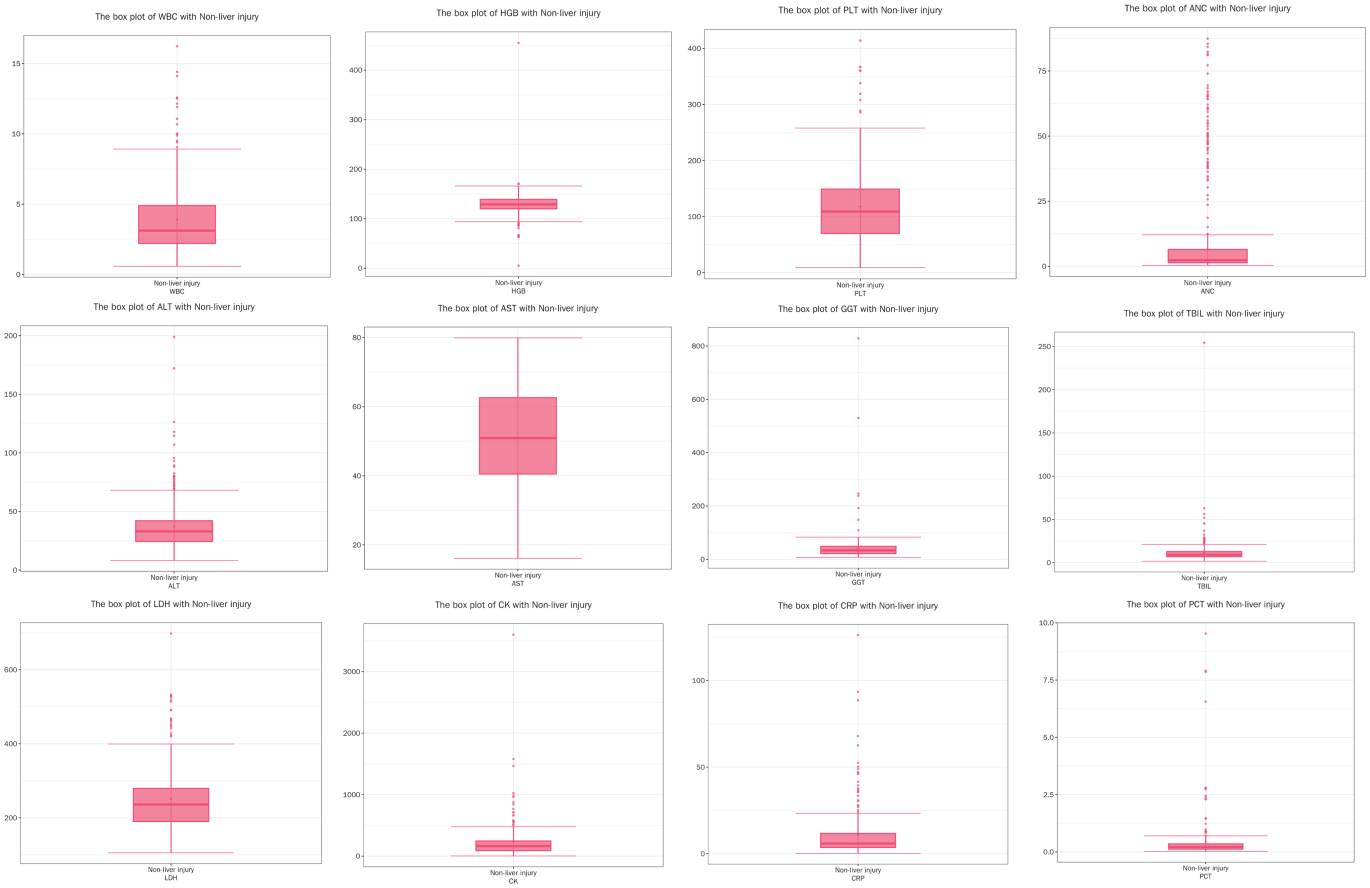
Boxplots of physiological and biochemical characteristics in non-liver injury group (median and interquartile range). Red line (median): represents the median, the middle value of the dataset. It divides the data into two equal parts, with 50% of the data points on either side. Circles (outliers): indicate outliers, which are data points located outside the ‘whiskers’ of the boxplot, typically beyond 1.5 times the interquartile range (IQR). These values may represent extreme data or errors, depending on the conte.

**Figure 2:**
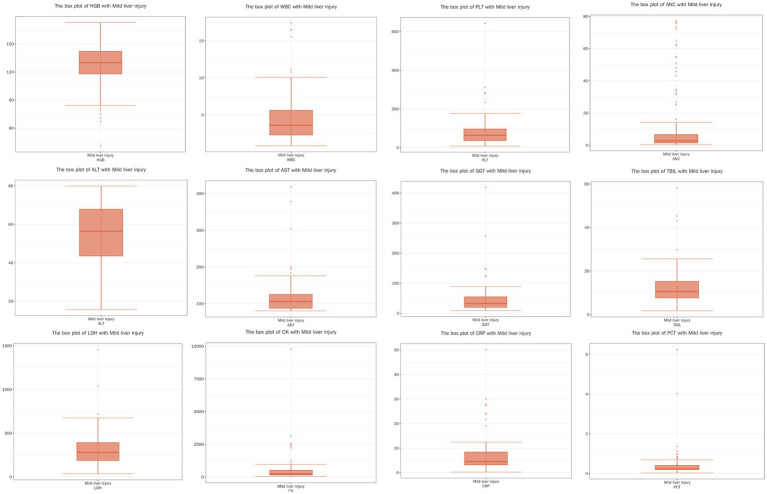
Boxplots of physiological and biochemical characteristics in mild liver injury group (median and interquartile range), red line (median): represents the median, the middle value of the dataset. It divides the data into two equal parts, with 50% of the data points on either side. Circles (outliers): indicate outliers, which are data points located outside the ‘whiskers’ of the boxplot, typically beyond 1.5 times the interquartile range (IQR). These values may represent extreme data or errors, depending on the conte.

**Figure 3:**
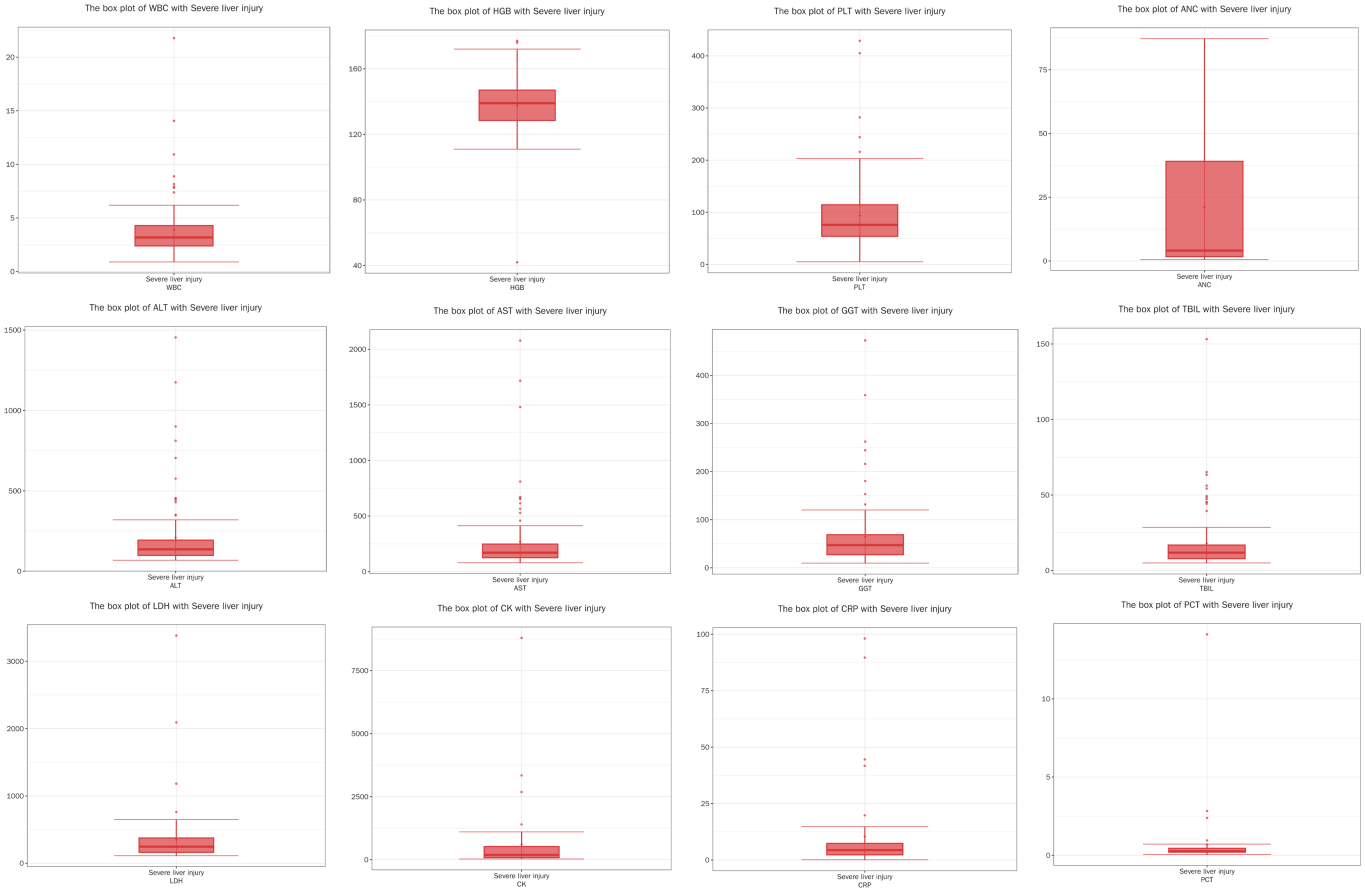
Boxplots of physiological and biochemical characteristics in severe liver injury group (median and interquartile Range). Red line (median): represents the median, the middle value of the dataset. It divides the data into two equal parts, with 50% of the data points on either side. Circles (outliers): indicate outliers, which are data points located outside the ‘whiskers’ of the boxplot, typically beyond 1.5 times the interquartile range (IQR). These values may represent extreme data or errors, depending on the conte.

Due to the non-normal distribution of several variables, as indicated by the results of the Shapiro–Wilk test, we opted to present these data using the median and IQR rather than mean values. The detailed data are presented in Table 3 and Figures 1–3. The median age of patients was 64 years (IQR: 51.00, 76.00). Laboratory parameters revealed notable findings, including a median WBC of 3.21*10^9/L (IQR: 2.28, 4.94). The median HGB level was 131.00 g/L (IQR: 121.00, 142.00). PLT showed a significant decrease with a median of 93.00*10^9/L (IQR: 60.00, 137.00). Liver function tests showed elevated ALT and AST levels, with medians of 41.60 U/L (IQR: 28.90, 69.20) and 65.90 U/L (IQR: 46.50, 103.70), respectively.

**Table 3:** Physiological and biochemical characteristics of dengue patients (median and interquartile range).

Variable	N	M (P_25_, P_75_)
Age (years)	533	64.00 (51.00, 76.00)
WBC (10^9/L)	533	3.21 (2.28, 4.94)
HGB (g/L)	533	131.00 (121.00, 142.00)
PLT (10^9/L)	533	93.00 (60.00, 137.00)
ANC (10^9/L)	533	2.60 (1.43, 9.90)
ALT (U/L)	533	41.60 (28.90, 69.20)
AST (U/L)	533	65.90 (46.50, 103.70)
GGT (U/L)	533	35.70 (21.50, 54.50)
TBIL (μmol/L)	533	9.60 (7.20, 13.90)
LDH (U/L)	533	243.30 (186.70, 322.20)
CK (U/L)	345	184.50 (95.50, 313.60)
CRP (mg/L)	372	5.30 (3.33, 9.98)
PCT (ng/mL)	371	0.22 (0.13, 0.36)

WBC, White Blood Cell Count; HGB, Hemoglobin; PLT, Platelet Count; ANC, Absolute Neutrophil Count; ALT, Alanine Aminotransferase; AST, Aspartate Aminotransferase; GGT, Gamma-Glutamyl Transferase; TBIL, Total Bilirubin; LDH, Lactate Dehydrogenase; CK, Creatine Kinase; CRP, C-Reactive Protein; PCT, Procalcitonin.

### Comparison of epidemiological features across the three groups based on liver injury severity

The age and gender distribution of patients in the three liver injury severity groups are summarized in Table 4. In the non-liver injury group, the largest proportion of patients were in the 71–80 age group (28.23%). In the mild liver injury group, the highest proportion of patients were also in the 71–80 age group (23.89%). The severe liver injury group had the highest proportion of patients in the 51–60 age range (21.84%).

**Table 4:** Age and gender distribution across liver injury severity groups in dengue patients.

Ages group	Non-liver injury			Mild liver injury			Severe liver injury		
	Cases (n/%)	Males (n/%)	Females (n/%)	Cases (n/%)	Males (n/%)	Females (n/%)	Cases (n/%)	Males (n/%)	Females (n/%)
18–20	7 (2.10)	5 (3.09)	2 (1.17)	4 (3.54)	2 (3.92)	2 (3.23)	3 (3.45)	2 (4.65)	1 (2.27)
21–30	10 (3.00)	5 (3.09)	5 (2.92)	5 (4.42)	4 (7.84)	1 (1.64)	12 (13.79)	5 (11.63)	7 (15.91)
31–40	20 (6.01)	13 (8.02)	7 (4.09)	8 (7.08)	4 (7.84)	4 (6.45)	11 (12.64)	7 (16.28)	4 (9.09)
41–50	32 (9.61)	11 (6.79)	21 (12.28)	6 (5.31)	2 (3.92)	4 (6.45)	11 (12.64)	2 (4.65)	9 (20.45)
51–60	69 (20.72)	27 (16.67)	42 (24.56)	21 (18.58)	6 (11.76)	15 (24.19)	19 (21.84)	10 (23.26)	9 (20.45)
61–70	50 (15.01)	28 (17.28)	22 (12.87)	19 (16.81)	6 (11.76)	13 (20.97)	12 (13.79)	6 (13.95)	6 (13.64)
71–80	94 (28.23)	48 (29.63)	46 (26.90)	27 (23.89)	13 (25.49)	14 (22.58)	16 (18.39)	9 (20.93)	7 (15.91)
81–90	46 (13.81)	23 (14.20)	23 (13.45)	23 (20.35)	14 (27.45)	9 (14.51)	3 (3.45)	2 (4.65)	1 (2.21)
>90	5 (1.50)	2 (1.23)	3 (1.75)	0 (0)	0 (0)	0 (0)	0 (0)	0 (0)	0 (0)
Total	333 (100.00)	162 (48.65)	171 (51.35)	113 (100.00)	51 (45.13)	62 (54.87)	87 (100.00)	43 (49.43)	44 (50.57)

### Comparison of physiological and biochemical parameters across the three groups based on liver injury severity

The comparison of physiological and biochemical parameters across the non-liver injury, mild liver injury, and severe liver injury groups was performed using appropriate statistical tests. Initially, the normality of the data was assessed using the Shapiro–Wilk test. As the data for several continuous variables were not normally distributed, non-parametric tests were used for comparison. Specifically, the Kruskal-Wallis test was applied to compare the median values of these non-normally distributed continuous variables across the three groups. A significant difference was defined as a *p*-value < 0.05, with the corresponding H-value indicating the strength of the difference between groups.

The results, as presented in Table 5, revealed significant differences in various physiological and biochemical parameters. For age, the severe liver injury group had a significantly younger median age compared to the non-liver injury and mild liver injury groups (*p* < 0.05). Regarding HGB, the severe liver injury group had significantly higher levels compared to the other two groups (*p* < 0.05). Additionally, Platelet Count (PLT) was significantly lower in the mild liver injury and severe liver injury groups (*p* < 0.05), consistent with the thrombocytopenia observed in more severe cases.

**Table 5:** Comparison of physiological and biochemical parameters across liver injury severity groups in dengue patients.

Groups physiological and biochemical parameters	Non-liver injury		Mild liver injury		Severe liver injury		H	*p*
	N	M(P25, P75)	N	M(P25, P75)	N	M(P25, P75)		
Age (years)	333	66 (54,76)	113	68 (55,78)	87	53 (37,67)	29.03	<0.05
WBC (10^9/L)	333	3.12 (2.21,4.9)	113	3.60 (2.30,5.61)	87	3.18 (2.39,4.29)	2.30	0.32
HGB (g/L)	333	129 (120,139)	113	130 (118,142)	87	139 (128.5,147)	20.65	<0.05
PLT (10^9/L)	333	109 (70,149)	113	64 (37,96)	87	76 (54,114.5)	51.96	<0.05
ANC (10^9/L)	333	2.33 (1.38,6.48)	113	2.74 (1.45,6.6)	87	4.06 (1.70,39.1)	9.27	<0.05
ALT (U/L)	333	33.1 (24.4,42.1)	113	56.4 (43.6,67.8)	87	13.65 (98.9,193.85)	276.40	<0.05
AST (U/L)	333	50.9 (40.5,62.6)	113	106 (87.7,125.1)	87	170.5 (125.80,248.25)	381.36	<0.05
GGT (U/L)	333	33.6 (21.4,48.6)	113	32.7 (20.1,54.7)	87	47 (27.2,68.8)	14.97	<0.05
TBIL (μmol/L)	333	9.3 (7,12.9)	113	10.6 (7.7,15.2)	87	11.8 (7.85,16.85)	12.37	<0.05
LDH (U/L)	333	236 (189.9,279.4)	113	279.9 (189.3,391.3)	87	247 (161.85,376.45)	10.65	<0.05
CK (U/L)	222	163.6 (90.43,274)	79	229 (153.45,501.95)	44	192.7 (82.38,523.08)	13.80	<0.05
CRP (mg/L)	244	5.9 (3.6,11.72)	82	4.48 (3.2,8.40)	46	4.39 (2.3,7.35)	8.94	<0.05
PCT (ng/mL)	240	0.21 (0.12,0.35)	82	0.23 (0.2,0.41)	49	0.26 (0.2,0.45)	12.67	<0.05

WBC, White Blood Cell Count; HGB, Hemoglobin; PLT, Platelet Count; ANC, Absolute Neutrophil Count; ALT, Alanine Aminotransferase; AST, Aspartate Aminotransferase; GGT, Gamma-Glutamyl Transferase; TBIL, Total Bilirubin; LDH, Lactate Dehydrogenase; CK, Creatine Kinase; CRP, C-Reactive Protein; PCT, Procalcitonin.

Liver function markers such as GGT and TBIL showed significant differences across the groups, with ALT and AST being the primary criteria for grouping the patients (*p* < 0.05). The severe liver injury group exhibited the highest levels of these markers, indicating substantial liver damage. Moreover, LDH and CK levels were significantly higher in the mild liver injury group compared to the other two groups (p < 0.05), reflecting greater tissue damage. Inflammatory markers, such as CRP and PCT, were also significantly higher in the severe liver injury group, suggesting a more pronounced systemic inflammatory response (*p* < 0.05).

### Comparison of clinical features across the three groups based on liver injury severity

The comparison of clinical features across the non-liver injury, mild liver injury, and severe liver injury groups was performed using the chi-square (X^2^) test for categorical variables. Pairwise comparisons were conducted using the adjusted *p*-values from the chi-square test. The results, summarized in Table 6, showed significant differences in the occurrence of some symptoms across the groups. These results indicate that while many symptoms were common across groups, significant differences were observed in bone joint pain and melena, highlighting their association with liver injury severity.

**Table 6:** Comparison of clinical features across liver injury severity groups in dengue patients.

Groups clinical features	Non-liver injury	Mild liver injury	Severe liver injury	X^2^	*p*
	N (%)	N (%)	N (%)		
Fatigue	307 (92.19)	103 (91.15)	83 (95.40)	1.36	0.51
Headache	179 (53.75)	58 (51.33)	43 (49.43)	0.6	0.74
Muscle pain	137 (41.14)	45 (39.82)	44 (50.57)	2.9	0.23
Bone joint pain	42 (12.61)	16 (14.16)	22 (25.29)	8.77	0.01
Poor appetite	296 (88.89)	109 (96.46)	80 (91.85)	5.66	0.06
Nausea	147 (44.14)	56 (49.56)	50 (57.47)	5.04	0.08
Dry mouth	212 (63.66)	70 (61.95)	52 (59.77)	0.48	0.79
Bitter taste in the mouth	155 (46.55)	59 (52.21)	39 (44.83)	0.1	0.95
Rash	119 (35.74)	43 (38.05)	33 (37.93)	0.28	0.87
Diarrhea	132 (39.64)	50 (44.25)	25 (32.18)	3.02	0.22
Hemorrhage	6 (1.80)	4 (3.54)	2 (2.30)	1.16	0.56
Melena	14 (4.20)	11 (9.73)	2 (2.30)	7.02	0.03

**Figure 4:**
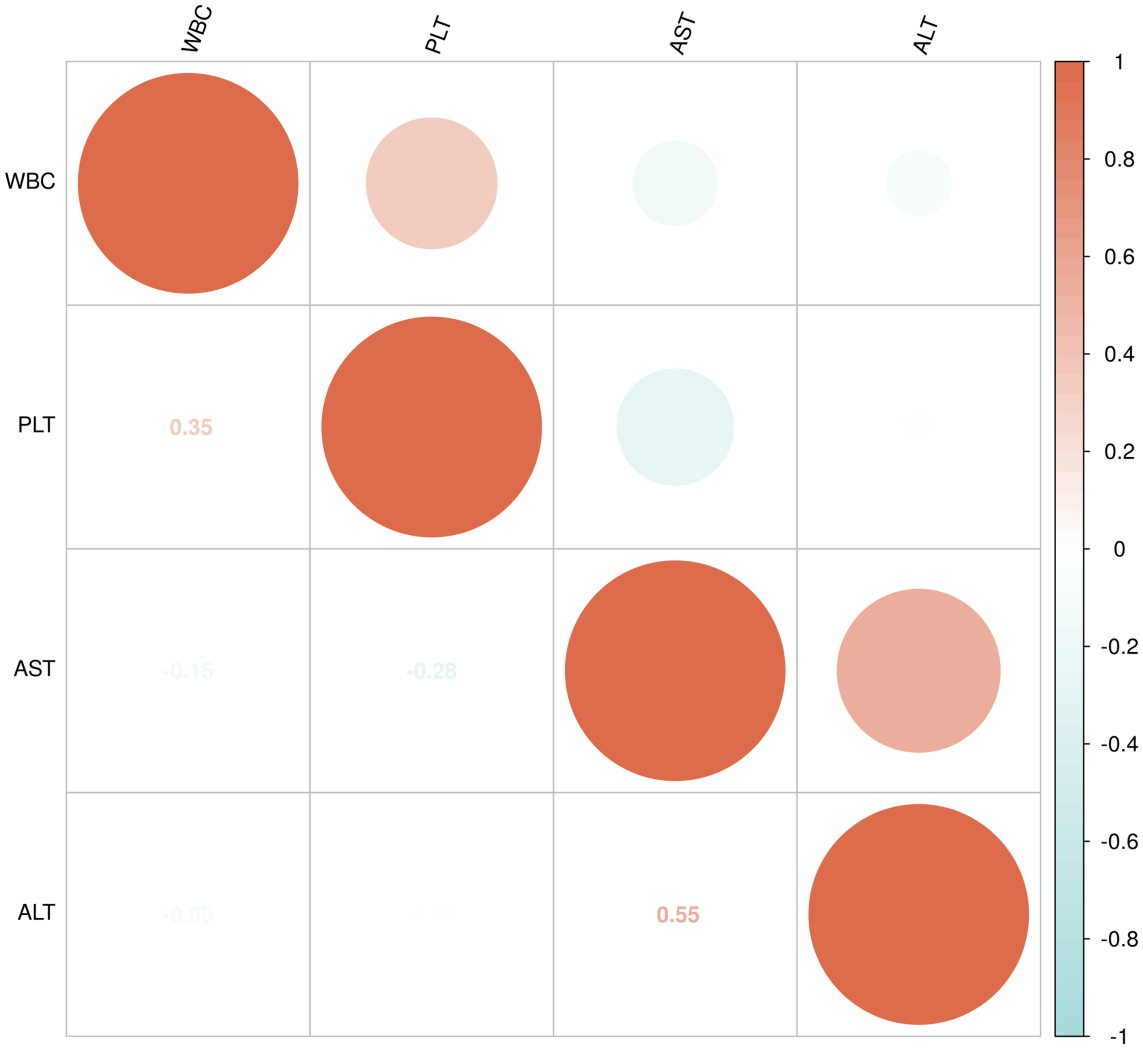
Correlation analysis of WBC, PLT, ALT, and AST in non-liver injury group.

The data for WBC, PLT, ALT, and AST in the non-liver injury, mild liver injury, and severe liver injury groups were found to not follow a normal distribution, so Spearman’s rank correlation was applied to assess the relationships between these variables (Figure 4).

For the non-liver injury group, a moderate positive correlation (0.35) was observed between WBC and PLT, suggesting a modest relationship. WBC showed a weak negative correlation with AST (−0.15) and ALT (−0.09), indicating minimal inverse relationships. Similarly, PLT showed a weak negative correlation with AST (−0.28) and a very weak negative correlation with ALT (−0.02). A moderate positive correlation (0.55) was observed between AST and ALT, indicating a tendency for higher levels of both enzymes (Figure 4).

**Figure 5:**
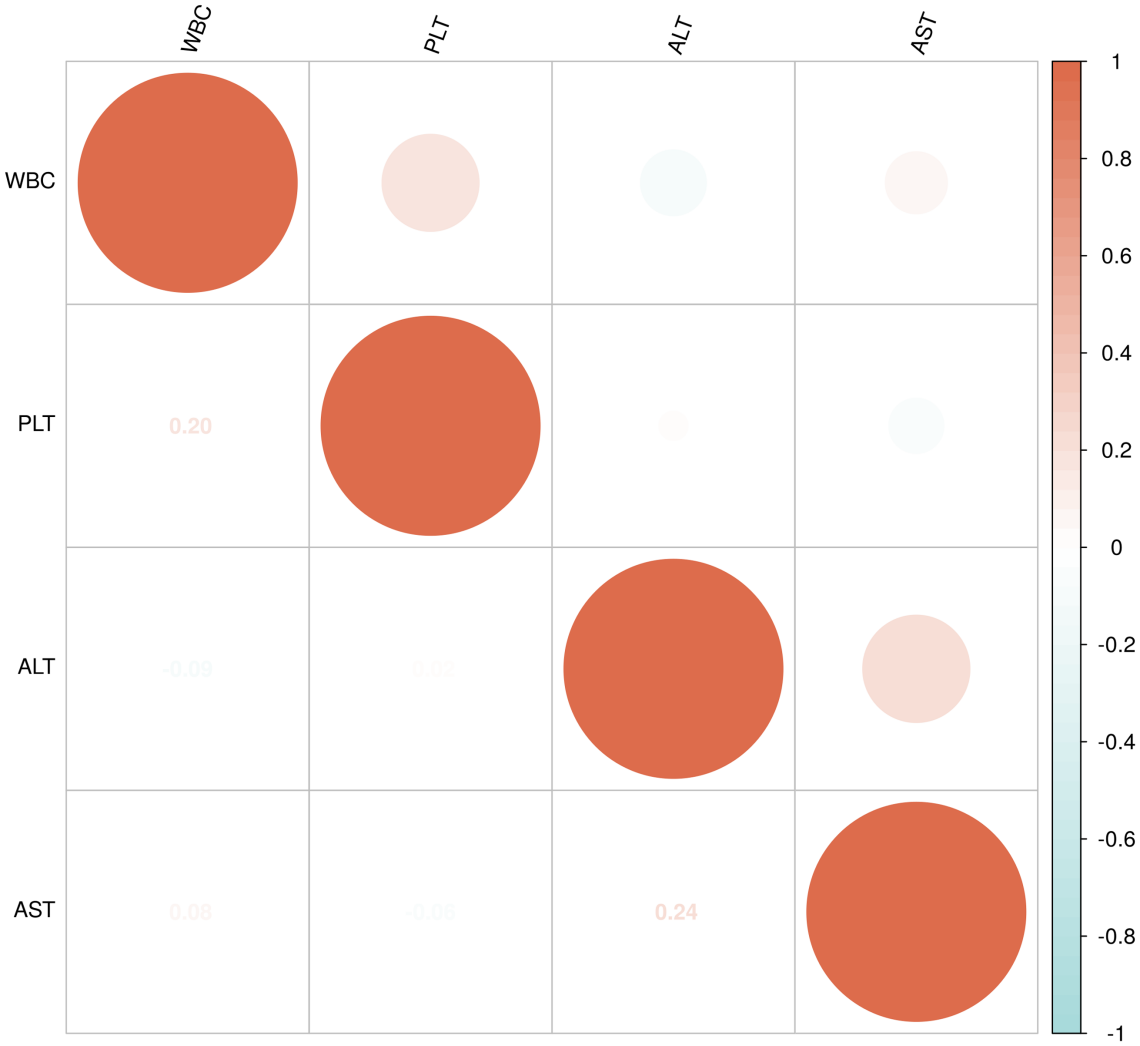
Correlation analysis of WBC, PLT, ALT, and AST in mild liver injury group.

In the mild liver injury group, WBC and PLT exhibited a weak positive correlation (0.20). WBC had a very weak negative correlation with ALT (−0.09) and a very weak positive correlation with AST (0.08). Similarly, PLT showed a very weak positive correlation with ALT (0.02) and a very weak negative correlation with AST (−0.06). A moderate positive correlation (0.24) was found between ALT and AST, suggesting a weak association between the two liver enzymes (Figure 5).

**Figure 6:**
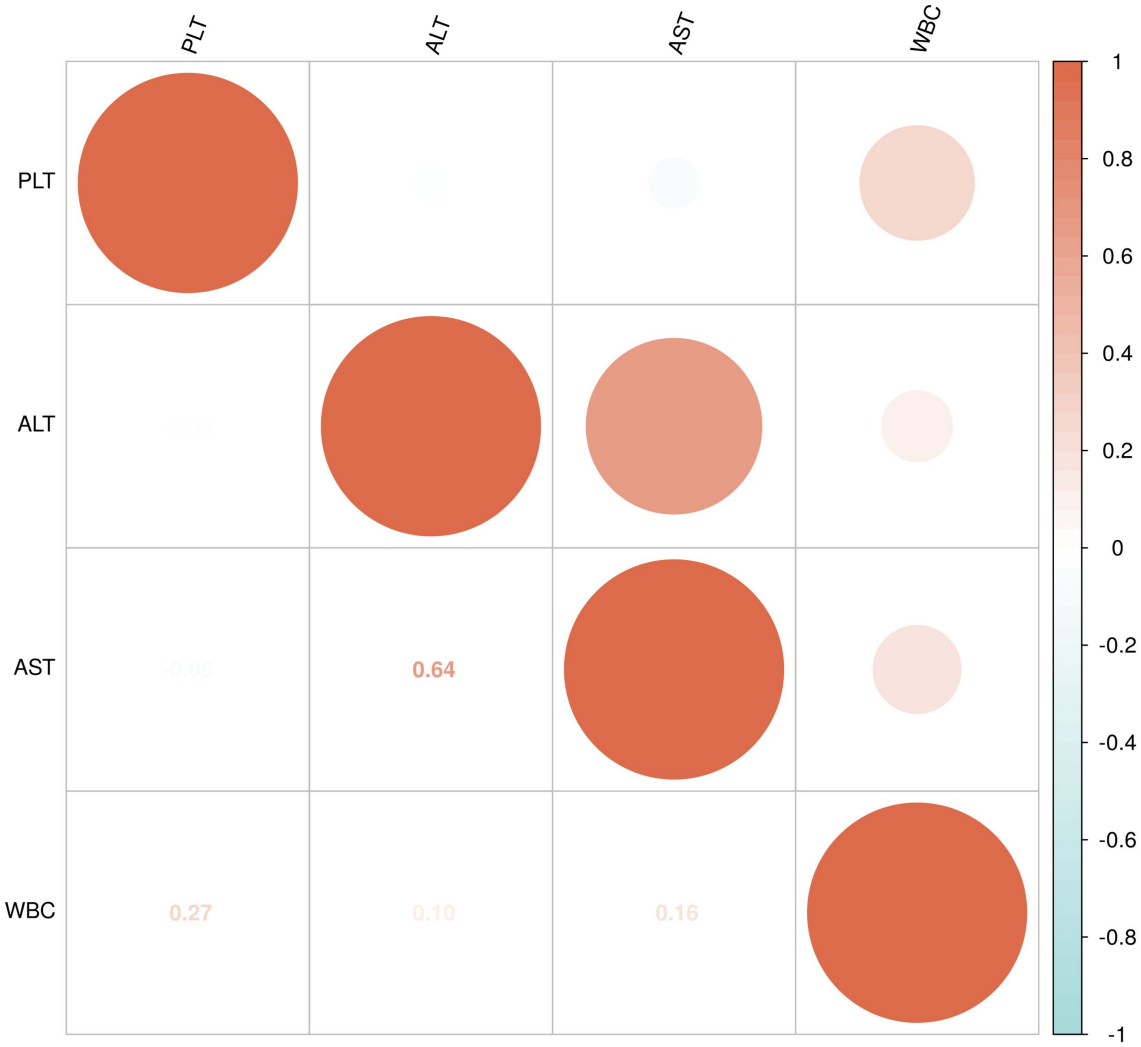
Correlation analysis of WBC, PLT, ALT, and AST in severe liver injury group.

For the severe liver injury group, PLT showed a very weak negative correlation with both ALT (−0.03) and AST (−0.05), indicating almost no relationship. A moderate positive correlation (0.64) was observed between ALT and AST, suggesting that higher levels of ALT are moderately associated with higher levels of AST. Additionally, WBC showed a weak positive correlation with PLT (0.27), and very weak positive correlations with ALT (0.10) and AST (0.16; Figure 6).

## Discussion

In this retrospective study, we analyzed the clinical features and laboratory indicators of 533 hospitalized dengue patients. The majority of patients were in the 51–80 age group, with a slightly higher proportion of females (51.97%) compared to males (48.03%). Significant laboratory abnormalities were observed, including thrombocytopenia (80.11%) and leukopenia (63.41%), which are characteristic of dengue infection. Liver enzyme levels were elevated in the majority of patients, with AST (84.99%) and ALT (52.53%) showing significant increases, indicating liver involvement. Elevated LDH and CK levels also suggested tissue damage, particularly in muscle tissues. Clinically, fatigue (92.50%), poor appetite (90.99%), and dry mouth (62.66%) were the most common symptoms, while melena was significantly more frequent in patients with mild liver injury. Additionally, the severity of liver injury was correlated with elevated liver enzymes, inflammatory markers, and indicators of tissue damage, highlighting the need for close monitoring of liver function in dengue patients. These findings emphasize the importance of identifying key laboratory indicators for early diagnosis and risk stratification of severe dengue cases.

The increasing incidence of dengue fever in China can be attributed to several key factors, including the rise in mosquito populations, climate change, urbanization, and enhanced population mobility. One of the most notable impacts of climate change on dengue transmission is the expansion of mosquito habitats. Aedes mosquitoes, such as *Aedes aegypti* and *Aedes albopictus*, thrive in warm and humid conditions, which are ideal for their breeding and growth. Global warming has not only expanded these mosquito habitats but also extended the active mosquito season, leading to increased opportunities for virus transmission (19). Climate change influences various environmental factors, including temperature, precipitation, and humidity. Rising temperatures accelerate the mosquito life cycle, especially the incubation and development of larvae, allowing them to breed and spread the virus more quickly. Increased rainfall further exacerbates the problem by creating more standing water, which serves as ideal breeding sites for mosquitoes. This is particularly evident in urban areas where rainwater often accumulates in uncleaned containers, thus contributing to higher mosquito populations and an increased likelihood of transmission (20–22). Studies have shown that tropical and subtropical regions are particularly vulnerable to dengue outbreaks, and with climate change, suitable mosquito habitats are expanding (23). In recent decades, many countries in Southeast Asia, Latin America, and Africa have experienced climate shifts that have facilitated the spread of Aedes mosquitoes into previously unaffected regions (24). Global warming is also predicted to extend the geographic range of dengue transmission to parts of Europe and North America, areas once considered low-risk regions (25, 26). Additionally, extreme weather events, such as heavy rainfall and heatwaves, intensify the risk of dengue transmission. Heavy rainfall not only increases mosquito breeding sites but may also lead to water contamination, putting further strain on public health systems (27, 28). These unpredictable environmental factors make dengue transmission increasingly unstable and difficult to control.

This study found that the number of individuals infected with dengue fever aged between 51 and 80 was higher than that of those under the age of 50. This could be attributed to the aging process, which weakens the immune system and reduces the body’s ability to mount an effective defense against infections (29). As a result, older individuals are more vulnerable to severe forms of dengue, including DHF and DSS, especially when they have pre-existing health conditions (30, 31). A study in Guangdong, China, examined severe dengue in elderly patients (≥65 years). Among 1,027 patients, 159 were diagnosed with SD, and 81% had comorbidities, primarily hypertension. Severe organ impairment (especially kidney damage) was the most common complication. Risk factors for severe dengue included chronic obstructive pulmonary disease (COPD), low red blood cell count, low serum albumin, and high fever. These findings highlight the importance of monitoring organ function and managing comorbidities in elderly dengue patients (32).

Our findings on liver involvement in dengue infection contribute new insights into the disease’s progression. While the elevation of liver enzymes such as AST and ALT is well-documented in existing studies, our study provides a more detailed analysis of liver injury in hospitalized dengue patients, specifically within the Chinese context. Our data confirm that liver enzyme levels, particularly AST, are elevated during the progression of dengue, and highlight the significance of these markers in predicting the severity of the disease. A meta-analysis has shown that both ALT and AST are commonly elevated in dengue patients, with abnormal AST levels observed in 80% of DHF patients and 75% of Dengue fever patients. This is consistent with the characteristics of impaired liver function in the 533 patients observed in our study (33). Additionally, A study found that ALT and AST levels increasing with the severity of the Dengue fever disease, although our study primarily relied on descriptive statistics to assess liver injury in dengue fever patients, previous studies have also highlighted that ALT and AST levels serve as valuable indicators of dengue severity (34). Moreover, studies have identified elevated AST levels within the first 72 h of fever onset as a significant predictor of severe dengue cases, with AST levels and platelet count serving as independent early markers for predicting the development of severe dengue. This also provides direction for future research. In subsequent studies, AST levels in patients should be regularly monitored, and the statistical significance of changes in AST levels in relation to disease progression can be analyzed (35).

Although liver function damage has been observed in dengue fever patients worldwide, there are relatively few reports on liver function impairment in dengue fever cases in China. Therefore, our research helps to address this gap in the literature. For example, Shubhransu Patro conducted a cross-sectional study and found that severe dengue patients exhibited significantly elevated ALT and AST, lower platelet counts (36). Sadia Khanduker conducted a cross-sectional study in Bangladesh to assess liver dysfunction in dengue patients, they highlighted that elevated ALT and AST were poor prognostic markers in dengue infection (37). An observational-descriptive study found that elevated ALT, AST, and prolonged APTT and PT were associated with severe dengue and higher mortality in children (38). Hina Saghir conducted a cross-sectional study in Islamabad found that elevated ALT levels were significantly associated with dengue hemorrhagic fever and dengue shock syndrome, and platelet count was correlated with elevated ALT levels (39). Ritin Mohindra conducted a retrospective observational study in North India and found that liver injury was significantly associated with dengue severity. Severe dengue patients exhibited markedly elevated AST, ALT, alkaline phosphatase, bilirubin, and CRP levels, with a strong correlation between CRP and liver enzymes (40).

Our study found significant liver injury in dengue patients, with elevated AST and ALT levels, which aligns with previous animal research. Sakinah et al. observed similar liver damage in BALB/c mice, including hepatocyte injury, sinusoidal widening, and mononuclear cell infiltration (41). Additionally, Paes et al. showed that DENV-2 replication in mice leads to hepatocellular necrosis, steatosis, and hemorrhage, with elevated AST and ALT levels correlating with liver damage severity (42).

In our study, 80.11% of patients exhibited thrombocytopenia. This condition is often associated with bleeding complications, such as petechiae, epistaxis, and, in more severe cases, gastrointestinal bleeding. Thrombocytopenia in dengue is caused by disrupted platelet production due to impaired megakaryopoiesis and thrombopoiesis, along with increased platelet clearance through activation, apoptosis, and enhanced removal. These combined factors lead to platelet depletion, contributing to thrombocytopenia in dengue patients (43). A study in Nepal found that leukopenia (64.68%) and thrombocytopenia (40.48%) were common in dengue patients. Both conditions were significantly associated with the severity of the disease, emphasizing their role in identifying progression to severe stages during the critical phase of dengue (44). Dengue fever exacerbates coagulopathy in patients, particularly those with pre-existing coagulation disorders, by inducing thrombocytopenia and downregulating key coagulation factors I, V, X, and XIII through the NS1 protein’s suppression of HNF4α, worsening the overall coagulopathy. Therefore, early treatment and preventive measures are essential for these patients to avoid the progression to severe dengue and its associated complications (45). Our study supports these observations and highlights the importance of early treatment and preventive measures to avoid the progression of thrombocytopenia to severe dengue and its associated complications. By integrating our findings with those from other studies, we emphasize the need for careful monitoring of platelet levels and coagulation parameters in patients with dengue, particularly in those with pre-existing conditions that may predispose them to more severe outcomes.

While this study did not collect data on patients’COVID-19 infection history or vaccination status, it is important to recognize the potential influence of these factors on the clinical outcomes of dengue. Recent research has indicated that COVID-19 infection and/or vaccination may have a significant impact on the susceptibility to, and severity of, subsequent viral infections, including dengue (46, 47). Additionally, COVID-19 vaccination has been shown to modulate the immune system in ways that could potentially interact with subsequent infections. Some studies have raised concerns that the immune response triggered by COVID-19 vaccines might alter the course of infections by other viruses. For example, reports have suggested that COVID-19 vaccination might increase the risk of symptomatic dengue infection by modifying the immune response, potentially increasing viral replication or immune activation (17, 48).

The COVID-19 pandemic has significantly influenced dengue transmission dynamics across various regions, with both direct and indirect effects on disease incidence, vector control efforts, and clinical outcomes. Studies from Bangladesh, China, Ecuador, Brazil, Thailand, and India highlight the complex interactions between COVID-19 interventions and dengue transmission. For example, in Bangladesh, a study on co-infection revealed that 31% of participants had both dengue and COVID-19, with severe health outcomes, including heart, brain, and kidney damage, being more common in the elderly (49). In contrast, strict COVID-19 measures in Guangdong Province, China, led to a dramatic decline in both local and imported dengue cases, with an estimated 6,557 cases prevented between 2020 and 2022, largely due to mobility restrictions and enhanced vector control efforts (50). Similarly, in Paltas, Ecuador, dengue transmission shifted from urban to rural areas during the pandemic, and there was a subsequent rise in cases post-pandemic, suggesting that mobility restrictions disrupted the usual urban transmission patterns (51). A study in Yunnan Province, China, noted that the reopening of borders after COVID-19 led to an increase in imported dengue cases (52). A predictive model in Fortaleza, Brazil, also showed a 72% reduction in dengue cases due to mobility restrictions, underscoring the significant role of human movement in disease spread (53). Meanwhile, in Thailand, dengue incidence decreased during the strict phase of the pandemic but rose again when preventive measures were relaxed, illustrating how changes in public health interventions directly impacted dengue trends (54). In addition to the general impact of the pandemic on dengue transmission, recent studies have also highlighted the clinical implications of dengue and SARS-CoV-2 co-infection, particularly in tropical regions. In Brazil, 6% of patients with confirmed COVID-19 were found to be positive for dengue IgM antibodies, indicating recent infection. Co-infected patients had significantly higher hospitalization rates (94.9%) and mortality (50%), with many requiring invasive mechanical ventilation. Secondary dengue patients with COVID-19 experienced more severe outcomes, including respiratory distress, longer ICU stays, and higher mortality compared to those with primary dengue infection (55). Similarly, in Vietnam, children with both infections presented with mild COVID-19 symptoms, but risk factors for severe disease included obesity, abdominal pain, and petechiae (56). In a study conducted on patients in dengue-endemic regions, it was found that both pre-existing SARS-CoV-2 infections and concurrent dengue infections significantly increased the incidence of severe dengue, including DHF and DSS. Specifically, co-infected patients showed higher occurrences of symptoms such as blood concentration, hypotension, thrombocytopenia, mucosal bleeding, and abdominal pain, which are key criteria for severe dengue. Patients with prior SARS-CoV-2 infections had higher incidences of thrombocytopenia, abdominal pain, and persistent vomiting, but without significant blood concentration or hypotension (57). Furthermore, while the co-infection did not have a significant impact on morbidity and mortality in all cases, the overlapping symptoms and laboratory findings of both diseases made it difficult to distinguish between the two. This highlights the importance of early diagnosis and the use of specific diagnostic methods to avoid misdiagnosis and improve patient management, especially in regions where both infections are prevalent (58). These studies underscore the need for careful clinical assessment and early detection of co-infection to improve patient outcomes and reduce mortality in regions affected by both diseases.

Recent advancements in the identification of predictive biomarkers have significantly enhanced our understanding of dengue fever and its progression, particularly in predicting the severity of the disease. As dengue continues to pose a major global health challenge, identifying reliable biomarkers is crucial for early diagnosis and timely intervention, particularly in severe dengue cases. Various studies have focused on exploring biomarkers, both established and novel, that can predict the outcome of dengue infections and help guide clinical decision-making. The study highlighted that oxidized HDL (oxHDL), in combination with other lipoproteins and inflammatory markers, may serve as a reliable prognostic biomarker for severe dengue fever. Elevated oxHDL levels were found to be significantly higher in patients with severe dengue compared to healthy controls, while conventional markers like HDL and LDL were lower in dengue patients (59). The study demonstrated that LDH levels at the time of diagnosis in dengue patients are strongly correlated with disease severity and can predict complications during the course of the illness. The linear correlation between LDH levels and disease severity was statistically significant, highlighting the potential of LDH as a reliable prognostic biomarker for assessing dengue severity and guiding clinical management (60). The study demonstrated that soluble interleukin-2 receptor (sIL-2R) levels are significantly elevated in patients with severe dengue compared to those with dengue with warning signs. The study identified a cutoff value of 5.379 ng/mL for sIL-2R, with a strong diagnostic performance for predicting severe dengue classification and hemophagocytic lymphohistiocytosis (HLH). This finding suggests that sIL-2R can serve as a useful predictive biomarker for severe dengue with moderate sensitivity and specificity, potentially aiding early surveillance and management in clinical practice (61). The study highlights the significant association between sphingolipids, particularly Sphingosine-1-phosphate (S1P), and the severity of dengue infection. It emphasizes the role of S1P metabolism in disease progression, plasma leakage, and overall virulence. The findings suggest that S1P and other sphingolipid-related enzymes, such as SMS1 and CERK, may serve as valuable biomarkers for predicting severe dengue (62). The study suggests that a panel of long non-coding RNAs (lncRNAs) has the potential to predict severe dengue with high accuracy. The predictive model, which demonstrated an AUC of 0.98, outperformed traditional warning signs in identifying high-risk patients, including those who later died. These findings highlight the potential of lncRNAs as reliable biomarkers for severe dengue, which could help reduce healthcare burdens and improve patient management in endemic regions (63). The study demonstrated that elevated levels of soluble tumor necrosis factor receptor 1 (sTNFR1) are predictive of subsequent hospitalization in outpatients with dengue virus infection. The findings suggest that sTNFR1 could serve as an important marker to guide clinical triage, helping to reduce the healthcare burden of dengue, especially in resource-constrained settings. sTNFR1 was also associated with longer hospitalization, IV fluid requirements, hemoconcentration, and thrombocytopenia, indicating its potential role in assessing disease severity and guiding patient management (64). The study emphasizes the significant role of soluble tumor necrosis factor receptor 1 (NST) in mediating the effect of NS1 to promote the progression of dengue severity. The findings suggest that NST is a better severity biomarker compared to NS1, as it can predict the progression from dengue without warning signs (DNWS) to dengue with warning signs (DWWS) and severe dengue (65). The study found a significant positive correlation between C-reactive protein and ferritin in dengue patients, suggesting that ferritin could serve as an additional biomarker, alongside CRP, for predicting hospitalization and assessing disease severity at an early stage of infection. The findings highlight the potential utility of ferritin as an early predictive marker for severe dengue (66).

In addition to the liver enzyme abnormalities, a weak negative correlation between platelet count and liver enzymes (ALT and AST) was observed in this study. Several studies have explored the relationship between these markers in dengue patients. The reduction in platelet count, commonly observed in dengue infection, is thought to be associated with viral-induced bone marrow suppression or increased platelet consumption due to immune activation. The weak negative correlation between platelet count and liver enzymes could reflect the complex pathophysiology of dengue, where liver injury may be coupled with thrombocytopenia in severe cases (43, 67). Interestingly, our study observed a weak negative correlation between platelet count and liver enzymes (ALT and AST) in dengue patients. While the precise mechanisms behind this relationship remain unclear, one possible explanation could be the role of oxidative stress in dengue pathogenesis. Previous studies have documented increased oxidative stress in dengue patients, which could influence both liver function and platelet production. Cherupanakkal et al. investigated the expression of endogenous antioxidant enzymes such as Catalase (CAT), Superoxide Dismutase (MnSOD), and Glutathione Peroxidase (GPx) in dengue patients, showing a significant down-regulation of these antioxidant enzymes throughout the course of infection. This down-regulation of antioxidant defenses could contribute to the redox imbalance, exacerbating both liver damage and thrombocytopenia. The study further suggests that the down-regulation of MnSOD expression during secondary dengue infections could be linked to more severe forms of the disease. This aligns with our findings, where oxidative stress may play a role in the observed correlation between platelet count and liver enzyme levels in dengue patients (68). In line with previous studies, our results suggest that liver enzymes (ALT and AST) and platelet count are critical parameters for predicting the severity of dengue. A study by Jean Pierre et al. utilized statistical and machine learning models to identify key clinical markers for distinguishing severe dengue (SD) from non-severe cases. They highlighted platelet count, along with abnormal ultrasound findings and lymphocyte count, as significant predictors during the febrile phase. Furthermore, elevated AST/ALT levels during the critical phase were strongly associated with severe dengue. These findings are consistent with our observations of the relationship between liver enzyme levels and platelet count, which may provide additional insights into the underlying pathophysiology of dengue (69).

In our study, we observed that patients with severe liver injury were relatively younger compared to those with non-liver or mild liver injury. This finding is intriguing and warrants further discussion. One possible explanation is that younger individuals tend to have a more robust immune response to viral infections, including dengue. While a strong immune response can be beneficial in controlling infections, it can also lead to more severe inflammatory reactions, such as cytokine storms, which might exacerbate liver damage (70–72). Moreover, younger individuals may experience immune enhancement due to secondary dengue infections, a phenomenon known as antibody-dependent enhancement, which has been associated with more severe clinical manifestations, including liver injury (73, 74). In terms of future research, it would be valuable to investigate whether younger individuals with severe liver injury experience a more robust inflammatory response and whether specific immune markers, such as cytokine levels, correlate with liver injury severity. Additionally, longitudinal studies with a larger cohort, including both primary and secondary dengue infections, could provide further insights into the role of immune response and the regenerative capacity of the liver in the pathogenesis of dengue-related liver injury.

While this study provides valuable insights into the diagnostic and prognostic markers of dengue, there are several limitations that must be considered. First, the retrospective nature of this study limits the ability to establish causality between the biomarkers (CRP, PCT, and WBC count) and the severity of dengue. A prospective study design would provide more robust data and better control for confounding variables. Second, the sample size, though sufficient to provide statistical significance, may still limit the generalizability of the findings to broader populations, particularly in regions with differing dengue epidemiology or healthcare infrastructure. A multi-center study with a larger, more diverse sample could help confirm the generalizability of the results. Third, while CRP, PCT, and WBC count are useful in assessing inflammation and infection, they are not specific to dengue infection. Elevated levels of these markers can also occur in other viral, bacterial, or inflammatory conditions, which may lead to false positives. Future studies focusing on dengue-specific markers and a more comprehensive set of clinical and laboratory parameters may enhance diagnostic accuracy. Another key limitation of this study is that it did not include pediatric dengue patients. This study has several limitations that should be considered when interpreting the findings. First, the analysis is limited to adults, which means that the results may not be directly applicable to children. Given the potential differences in disease progression, biomarkers, and treatment responses between adult and pediatric populations, further research involving children is essential to fully understand the scope of dengue and its management across all age groups. In fact, Deng et al. reported a significant increase in the global burden of dengue in children and adolescents, with dengue-associated disability-adjusted life-years (DALYs) rising from 910,458.60 in 1990 to 1,059,428.31 in 2021, a 16.36% increase. Additionally, the age-standardized DALYs rate per 100,000 population increased from 40.17 in 1990 to 41.50 in 2021. These trends underscore the growing burden of dengue among children and adolescents, particularly in regions with middle socio-demographic index levels, where the disease burden remains highest (75). This highlights the need for further studies focusing specifically on pediatric dengue cases. Furthermore, as a retrospective study, we faced limitations related to the availability and completeness of patient data. The study was limited by the absence of comprehensive follow-up data, particularly regarding long-term clinical outcomes. This hindered our ability to assess the full spectrum of disease progression and recovery, especially with respect to long-term complications associated with severe dengue. Additionally, we were unable to accurately determine the mean onset of fever days across all participants, as inconsistent reporting or missing data regarding the exact day of fever onset impacted our analysis. As a result, we could not assess the precise duration of the febrile phase in relation to disease severity. Other limitations include the underrepresentation of certain subgroups, such as pediatric patients, and the lack of analysis based on viral serotypes, which could influence disease outcomes. Lastly, although we aimed to gather data from a large cohort, the retrospective nature of the study limits our ability to establish causal relationships between clinical features, laboratory markers, and disease severity. Future prospective studies with more rigorous data collection methods and longer follow-up periods are needed to confirm and build on our findings. In conclusion, while this study provides valuable insights, the aforementioned limitations highlight the need for further research to validate the findings and improve diagnostic and prognostic strategies for dengue management, especially in pediatric populations.

## Conclusion

This study provides a comprehensive analysis of the clinical features and laboratory indicators of 533 hospitalized dengue patients in China. The findings highlight several key aspects of dengue infection, including the prevalence of common symptoms such as fatigue, poor appetite, and dry mouth, as well as significant laboratory abnormalities like thrombocytopenia, leukopenia, and elevated liver enzymes (AST and ALT), which are indicative of liver involvement. The severity of liver injury was associated with higher levels of liver enzymes, inflammatory markers (CRP, PCT), and tissue damage markers (LDH, CK), underscoring the importance of closely monitoring liver function in dengue patients. The study also revealed that thrombocytopenia and elevated liver enzymes, particularly AST and ALT, serve as critical biomarkers for assessing the severity of dengue and predicting potential complications. Moreover, the findings emphasize the need for early diagnosis and risk stratification based on laboratory markers. This information can aid in guiding clinical decision-making and providing timely interventions for patients at risk of developing severe dengue manifestations.

In conclusion, the results of this study underscore the importance of identifying key clinical and laboratory markers for early identification of severe dengue cases, enabling improved patient management and outcomes. Further research, including prospective studies and multi-center trials, is warranted to validate these findings and explore additional biomarkers for effective prediction and management of dengue disease severity.

## Data Availability

The original contributions presented in the study are included in the article/supplementary material, further inquiries can be directed to the corresponding authors.

## Footnotes

^1^ https://worldhealthorg.shinyapps.io/dengue_global/
